# Loneliness of older black South African women subjected to forcible relocation

**DOI:** 10.1080/16549716.2019.1672329

**Published:** 2019-10-09

**Authors:** Vera Roos, Norah Keating, Carlien Kahl

**Affiliations:** a Optentia Research Focus Area, North-West University, Vanderbijlpark, South Africa; b Centre for Innovative Ageing, Swansea University, Swansea, UK

**Keywords:** Black older women, loneliness, South Africa, social exclusion, remedies for loneliness

## Abstract

**Background**: A cohort of older black South African women, forcibly relocated during apartheid, has grown old in these places. Even after 50 years, residents in a rural township expressed no connection to place and ruptured intergenerational relations. Their sense of community was based almost exclusively on their links with others who shared their history of relocation.

**Objective**: This article seeks to understand loneliness of a group of older women who have been rendered vulnerable by longstanding exclusion from community, services and material resources. We use loneliness as a metric for exclusion from social relations.

**Methods**: Sixteen Setswana-speaking women in Ikageng, a township in North West Province of South Africa (age 61–73), participated in the Mmogo-method® and open-ended interviews. Textual data were analyzed using thematic analysis, visual data analysis of elements and symbolic representations of loneliness.

**Results**: Loneliness is a powerfully unpleasant experience of not being able to interact with other people in general, or more specifically as a result of the loss of particular people (including spouses, parents and children) and isolation provoked by the impact of relational interactions and group dynamics. Loneliness was mitigated by socializing and gathering for traditional activities, performing spiritual rituals, and keeping busy individually or with others, thus reinforcing a core theme that any social interaction alleviates loneliness.

**Conclusions**: Even though loneliness is powerfully unpleasant, it is an expression of the importance of social interactions formed in a particular context. In the face of longstanding societal exclusion and disconnection from community, social connections are central to identity and to survival.

## Background

Loneliness among older adults has figured increasingly prominently on international research and policy agendas. North America has seen national campaigns initiated by governments and non-governmental organizations (NGOs) to end loneliness [1,2]. In Europe, projects have been launched to identify risk factors for loneliness and to suggest strategies to enable older people to be less isolated and lonely [3,4]. The UK has highlighted the issue by appointing a Minister for Loneliness in an attempt to develop a national strategy to combat loneliness [5]. Clearly, there is consensus throughout the global north that addressing loneliness is important. There is evidence from those regions that measures to counter loneliness have buffering effects against poor health and mortality outcomes if they are effective [6–9].

Despite such international visibility, loneliness has not found a place on national policy, advocacy or research agendas in sub-Saharan Africa. We believe that increasing the understanding of loneliness in the region is important. One of the pressing initiatives is the Sustainable Development Goal to leave no one behind [10]. This is particularly relevant in the context of pre-1994 South Africa when apartheid brutally removed all people of color to the margins of society [11,12]. Despite efforts post-1994 to redress the damage, there is still a glaring lack of attention to older black persons’ mental health needs or service provision [13]. The purpose of this paper is to begin to address this agenda by presenting findings from an exploration of the meaning loneliness holds for a group of marginalized older women in South Africa.

### Assumptions about loneliness experiences

This study adopts two assumptions about context. First, human experiences are formed and informed by the socio-cultural, economic, political and historical contexts particular to that time and place [14,15]; and second, loneliness is to be viewed in relation to interpersonal contexts.

From a social-ecological perspective, loneliness as a subjective experience develops in relation to the micro, meso and macro environments in which people function [16]. This acknowledges diversities found among populations even though they share the same geographical context [16]. Social contexts are relevant for loneliness in terms of the discrepancy between what individuals desire in a personal network of relationships and what they experience [16,17]. The validity of such views of loneliness has been proven robust over almost 30 years of research, mostly in Western societies [18–20]. Research also provides evidence that social relationships can help people navigate the challenging life circumstances that accompany later life [16,21–23]. Loneliness in relation to interpersonal contexts has been researched in terms of interpersonal styles that fail to address psychological needs for confirmation [24].

### Contexts of loneliness in post-apartheid South Africa

The group of black South Africans in this study grew up and have grown old within a nationally implemented policy of racial segregation in the apartheid period (1948–1994). It represented discriminatory social engineering on a grand scale, whereby thousands of people were re-organized according to racial divides and forcibly removed from their homes to unfamiliar areas during the 1960s. The execution of apartheid legislation, influenced social life in general, racial group relations in particular, and impacted the daily relational interactions between people of different ages [25,26]. In accordance with the Group Areas Act [11], the Setswana-speaking people in this study were forcibly relocated from their homes in Kloppersville, a racially integrated community to Ikageng, a poorly resourced area in North West Province, South Africa. Even after 50 years, their sense of community remained based almost exclusively on their links with others who shared their history and who provided safe interpersonal spaces [26]. The question driving this study is how a group of older black women forcibly relocated in South Africa during apartheid experiences loneliness.

## Methods

A critical-realist ontology underpins this qualitative study and informs the notion that multiple, subjective perspectives of older individuals reveal patterns or indications of loneliness as social reality [27–29].

### Procedure and participants

Ethical approval was obtained from the North-West University’s ethics committee. The research team consisted of two qualitative researchers with basic knowledge and understanding of Setswana, and four postgraduate first language Setswana-speaking students in psychology who were trained as field workers and translators. Data were collected at the Day Care Centre for the Aged in Ikageng and at the house of a volunteer in the community.

Our sampling approach most closely approximates purposive sampling. Following Etikan et al. [30], we assumed homogeneity related to the phenomenon of interest - all older people in Ikageng being at high risk of loneliness. All had had their lives powerfully shaped by the racial segregation of apartheid, by their experience of forced relocation and by 50 years of living in a desolate, resource-poor place. Participants were selected based on their willingness to take part in the study. Recruitment was done by the manager of the day centre and a key community member. In total, there were sixteen participants, aged 61–73.

Yet, while we assumed that all older members of the community were at risk of loneliness, only two men agreed to participate. The small number of men in the study meant that we were not able to engage with the question of possible gender differences in their experiences of loneliness. We therefore, present data from the 16 women participants.

Participants were divided in two groups of seven and nine people per group. They took part in the Mmogo-method®, a projective visual data-collection method [31–33]. Subsequently, 15 women volunteered for individual interviews.

### Data gathering

The Mmogo-method® according to Chilisa [34], is an example of an Afrocentric methodology that is applied in four phases. In Phase 1, context was created by introducing the topic, researchers and participants, obtaining informed consent and explaining the rules of engagement. In Phase 2, participants were invited to seat themselves in a circle. They were provided with malleable clay, beads of different sizes and colours, dried grass stalks and a round cloth and were requested to construct representations of how they experienced loneliness. Taking into consideration that the older adults might feel exposed if expected to share their experiences of loneliness in the presence of younger Setswana-speaking people, the prompt to stimulate their visual constructions was formulated as a request to learn about loneliness from them. Participants completed the constructions in about 45 minutes, and each explained in turn what they had made. In Phase 3, participants explained their visual representations in no particular order, following a participant who volunteered first. The group was invited to augment their accounts. Researchers verified their understanding of the explanations offered. Finally, in the last phase, participants reflected on their experience which served as debriefing. They joined in refreshments with the researchers and departed. On completion of the data collection, researchers also engaged in debriefing conversations. All discussions were audio recorded, translated and back translated, thus serving as textual data. Photographs of the visual presentations were used as visual data.

Trained field workers conducted open-ended individual interviews of 45–60 minutes with participants. Three open-ended prompts were used: What can we learn from you about loneliness? Help us to understand what contributes to loneliness. What can help to counter loneliness?

### Data analysis

Textual and visual data were analysed separately by two independent coders using ATLAS.ti version 8, and by applying inductive thematic analysis [35].

#### Textual data

Researchers read line by line to familiarize themselves with the data and to generate initial codes. Searching for themes involved collating codes and gather all data relevant to the theme. Themes were revised and discussed until researchers reached consensus, and clear definitions and names were formulated [35].

#### Visual data

Following Roos and Redelinghuys [36], the visual representations were studied to obtain a first impression of what was portrayed and what contexts represented. Visual elements were described in terms of form, composition and placement and symbolic meanings attributed by participants were added. Finally, the significance of the visual presentation in terms of the broader research question was analyzed.

### Trustworthiness

To ensure trustworthiness, principles and strategies of crystallization described by Ellingson [37] were used. Multiple perspectives of explanations were obtained from the textual and visual data. Co-analyses of visual and textual data and researchers’ reflexivity added to the rigor of the data [37,38]. Credibility was further enhanced by member checking in the course of data collection. Transferable knowledge about loneliness experiences, components of loneliness and strategies to deal with it was identified and supported with participants’ direct quotes.

## Results

The participants cast loneliness as a powerful and negative response to the loss or absence of others and to being alone. A striking presentation is the profound, often visceral experience that participants call loneliness.

‘Loneliness is a thing of being alone, even if its five minutes without someone, your heart worries [if] everything’s ok. It is a feeling of being under “attack”’ (B10, interview).

‘You feel painful and remember the late people and feel depressed. You feel bitter because you are alone, thinking you need comfort, it brings sorrow’ (B1, interview).

‘When you are hurt and there is none to comfort you. You wish you were dead’ (B8, interview).

‘When lonely, you [are] just like a lost person (gasping her breath). You have feelings like the kind of life you are living is not real’ (B16, interview).

The detailed analysis of loneliness is presented below. Table 1 presents a summary of the two main themes: components of loneliness and remedies for loneliness.

**Table 1.** Themes and subthemes of loneliness.

Components of loneliness	Loss of specific relationships Not being able to interact with anyone Impact of painful interactions
Remedies for loneliness	Anyone will do Socio-cultural and spiritual rituals Keeping busy: alone or with others

### Components of loneliness

Three types of experiences contribute to loneliness: (1) loss of intimate relationships to particular people; (2) not being able to interact with people; and (3) impact of painful interactions.

#### Loss of specific relationships

Loneliness is expressed in relation to losing a spouse; through death, divorce or separation [Participant 6, Mmogo-method; B1, B2, B6, B9, B15, interviews]. The response by Participant B7 [interview] serves as illustration: ‘Now (that) I’m old and alone, there is no old man who can shout in the house, to take out loneliness’.

Loneliness is also associated with the loss of parents or of children: ‘I can see (experience) that there is loneliness and you also think of those who have passed away – our parents. You think [what] if they were [still] here’ [Participant 10, interview]. The painful loss of her son is described by Participant B4 [interview]:

I had a son, a policeman. When he came he will say “Mama (mother), are you still on that machine holding the needle?” (doing needlework). He then rubbed [my back]. When he leaves, I feel lonely and think about him. When he visits and found that I am not here, he leaves a box of Kentucky and R100 covered with a cloth on my bed. My son had long died. So I am feeling very lonely. I think about him all the time, wishing that I could have somebody to talk to.

#### Not being able to interact with anyone

**Figure 1.**
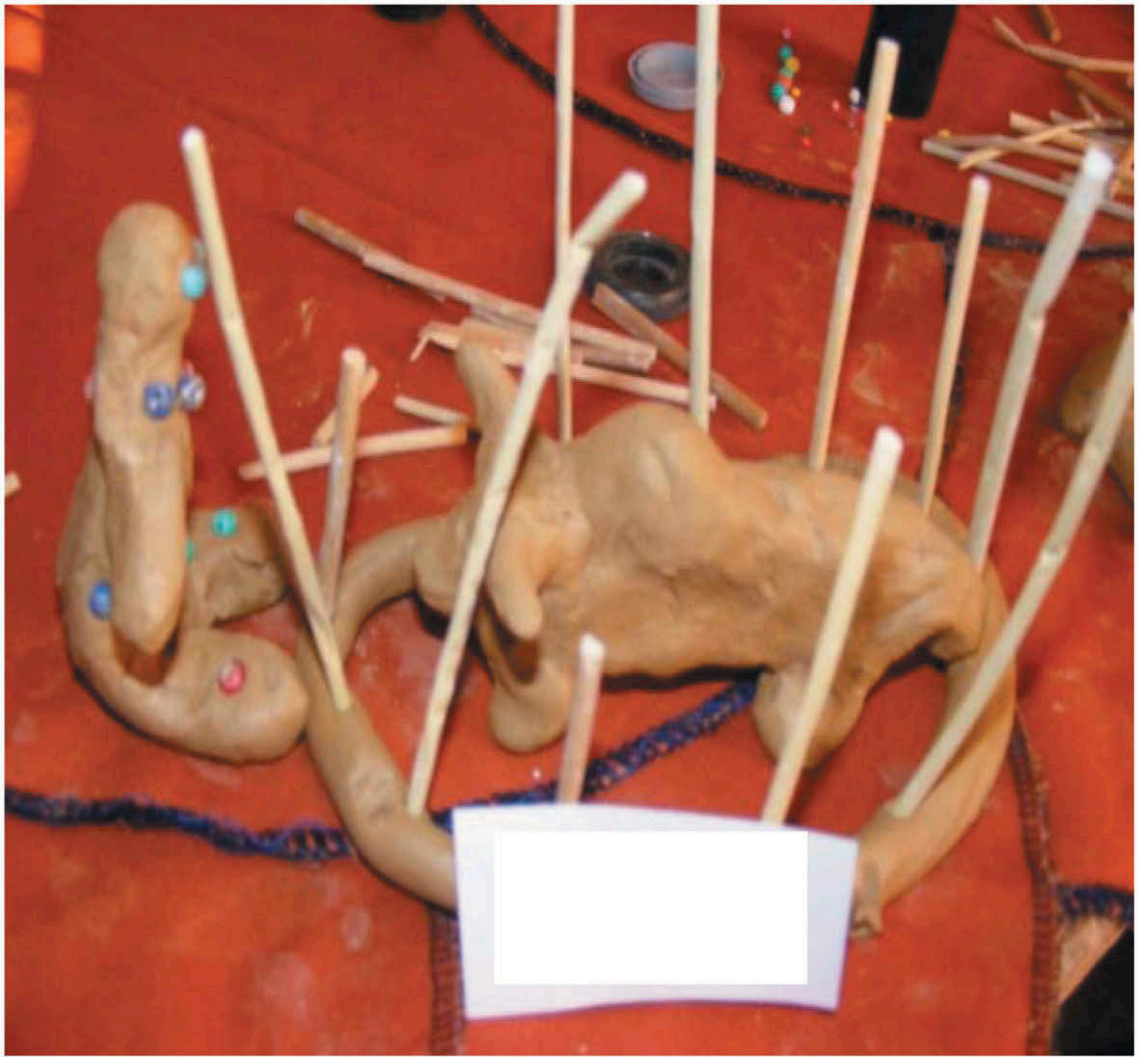
Loneliness as experience of being excluded.

A recurrent theme was that loneliness occurs ‘when you don’t interact with people’, Participant B8 [interview] explained; or in the words of Participant B7 [interview]: ‘Loneliness is when you are unable to go to other homes, in other homes to other people’. Unable to interact or being excluded from social interaction means to be excluded from participation, which is vividly portrayed in Figure 1. A woman [Participant 6, Mmogo-method] depicts herself sitting alone as an outsider, watching a cow confined in a *kraal* (pen where animals are kept) to convey that loneliness is the inability to participate.

**Figure 2.**
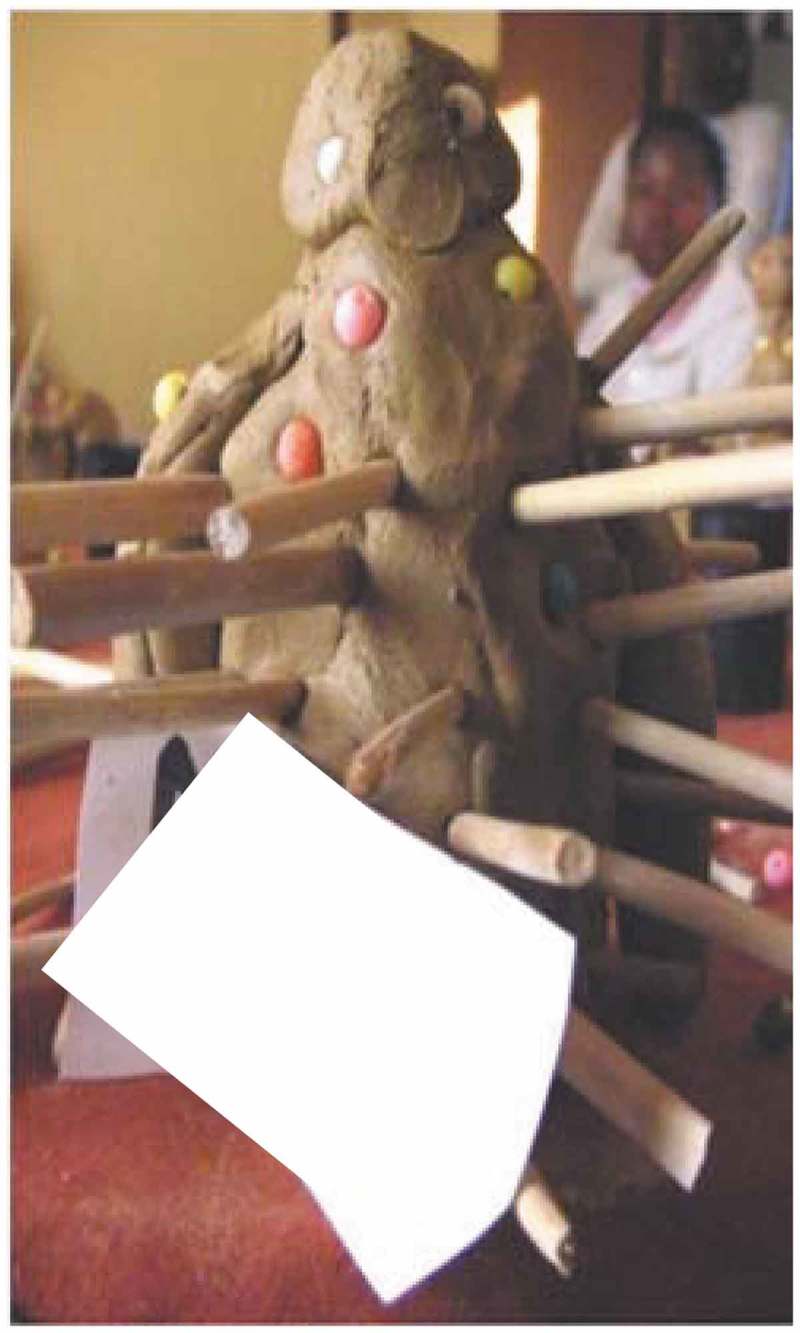
Older woman suffering from arthritis.

People are excluded from interacting for different reasons, such as ill-health. In Figure 2, Participant 4 [Mmogo-method] shows that stabbing pain from arthritis causes her to withdraw from people until she feels better:

I then lie down until the worst passes. I feel I can contribute more to the people around me than while I’m in pain. Then, when I’m among other people, I experienced that the pain and loneliness lift away leaving me relieved.

Sometimes there is nobody to interact with and older women who are bedridden lack the mobility needed to seek company: ‘Like me, I cannot go anywhere because of my knees. I feel lonely’ [B3, interview]. Participant B1 [interview] says: ‘There is no one to keep you busy’. The lack of people to interact with impacts negatively on older participants’ motivation to care for themselves: ‘If I am alone I become lazy so that I cannot make myself even soft porridge. Loneliness then comes back to you as I can’t see myself drinking tea alone’ [B4, interview].

#### Impact of painful interactions

Paradoxically, interactions with others also can lead to a sense of loneliness and exclusion [39–41]. A woman reflected on the impact of relational interactions between them, as domestic workers, and their usually white employers: ‘Even when you are working as a domestic worker for a white lady and she says to you that you are like this or that, you don’t get fed up that time. When you are alone, it is then when you feel the pain’ [B8, interview]. Participants also described the negative impact as a result of interacting with people from a different community: ‘Sometimes you visit other people. You go there and you greet them and you stay and relax at their place. They think you’ve come to eat their food’ [B10, interview].

### Remedies for loneliness

Participants in this study used three main strategies to deal with loneliness: interacting with anyone who is available; engaging in socio-cultural and spiritual rituals; and keeping themselves occupied.

#### Anyone will do

Loneliness is alleviated when with other people: ‘If you want to do away with loneliness you need to be in the company of people’ [B9 Interview]. They interact for the purpose of socialization, to acquire new perspectives, to be cared for and to forget personal troubles, as illustrated in these comments:

‘If you go out and socialize, you learn new ideas and forget your troubles’ [B8, interview].

‘ … forget all sorts of things that make you lonely and you become happy again’ [B7, interview].

‘Emotions become relieved and it settles*’* [B3, interview].

For the most part, the interactions do not have to serve a specific purpose or need in order to take place. Participant A2 [interview] explains: ‘If loneliness attacks me, I visit someone to chatter or someone should visit me and share ideas, not bad things or issues, but nice and funny [things] for us to laugh’. Participants thus engage in activities with others by gardening, knitting together, or talking (sharing ideas), singing hymns, passing jokes, or just laughing together [Participant 6, Mmogo-method]. However, not all participants were of this view. Participant B10 [interview] explained that she doesn’t like noise: ‘Sometimes they make noise, to that extent that I leave the house and go and stay alone. So that I listen to my mind’.

#### Socio-cultural and spiritual rituals

**Figure 3.**
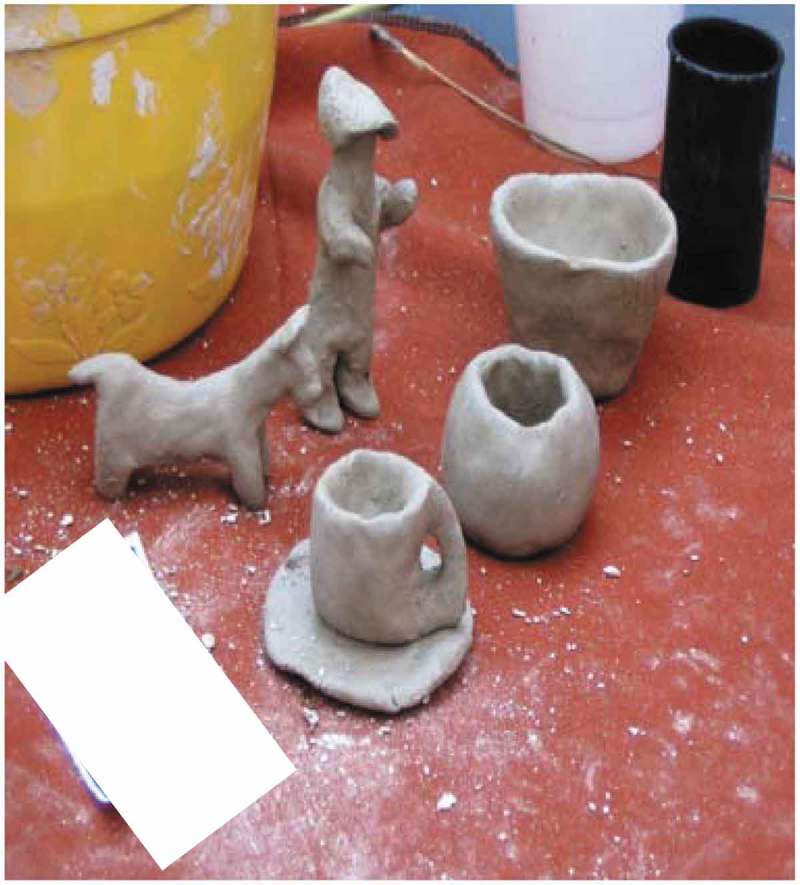
Traditional practices: making beer and socializing.

Socio-cultural rituals during weddings, funerals and traditional practices such as beer making offer older adults a buffer against loneliness: ‘Weddings of family members also help a person not to feel lonely because there is talking, singing and dancing’ [B12, interview]. Many of the women created traditional beer pots called *moritshana*. Beer making forms an integral part of socializing and teaching younger family members traditions, as illustrated in Figure 3.

Participant 5 [Mmogo-method] says:

The pot of Tswana beer represents the traditional Tswana culture. We make beer, come together and pass jokes while drinking and socializing so that we don’t feel lonely.

Spiritual rituals as a remedy for loneliness were also highlighted. These included going to church, where there is the opportunity to sing and dance and to talk to people to ‘heal you’ [B1, B3, B7, B8, B9, B10, B15, B16 interviews; P3, P7, P9, Mmogo-method].

#### Keeping busy: alone or with others

All participants highlighted the importance of getting into action and doing something to deal with loneliness and its impact. ‘The remedy is to go out and do something’ (B8, interview). They suggest: cleaning the house, yard, doing washing, baking, getting a spade and digging, singing hymns and visiting sick people [A2, B1, B4, B6, B10, interviews]. Joining a group to share in communal activities, is also proposed: ‘Ikageng Centre for the Aged is a gathering place for older persons to get together and do different things’ [Participant 6, Mmogo-method]. However, participants reported that a lack of money impacted on their ability to keep themselves busy.

## Discussion

Loneliness is a powerfully unpleasant feeling, compared to being under attack, particularly when alone and prevented from being with anyone. Loneliness is strikingly associated with profound visceral experiences, accompanying the subjective negative feelings, such as feeling sad and neglected [42,43]. The unbearableness of the bodily and emotional experience of loneliness sets in motion different strategies to deal with it.

This group of black older South African women illustrates that the loss of a significant close relationship with a spouse contributes strongly to loneliness [43], as does the loss of any meaningful relationship in which they experienced care and protection, thus raising the possibility that loneliness may be attributed to any significant relational loss. Contrary to findings that have linked loneliness to the subjective assessment of a deficient number of social relations and a lack of intimacy quality [44], this study indicated that loneliness is experienced when there is nobody to interact with, regardless of the closeness of the relationships. Social interaction may be impossible due to ill-health or as a result of withdrawal from contact with others. Isolation in turn reduces the likelihood of developing new perspectives, or moving from a self-centred to an other-centred perspective, or from receiving confirmation from others. Potential consequences of social isolation and the associated unbearable feelings add urgency to the need to take steps to ensure that ‘loneliness does not find a place to stay’ [B8, interview].

The findings support previous research showing that older adults who have become isolated or have experienced social isolation for many years are especially prone to feel lonely [45]. However, in this context, when in the company of others, the women felt energized and able to provide care for themselves and for others. Their references to interacting with anyone, irrespective of the quality of the relationship or the nature of the interactions, could imply that this group of women has a low ‘loneliness threshold’ [46, p.105]. However, the socio-cultural context offers a more fundamental possibility that to be human means that individuals see their existence in their relationship with others [34,47].

A unique component revealed in this study is that loneliness is associated with painful interactions – either on an individual or group level. In the socio-political context of South Africa pre-1994, many black people were forced to assume a submissive position in relation to white people who frequently employed them as domestic workers. Many were subjected to humiliation, without legal or social protection and that inflicted shame. Individuals also experienced a painful impact from interacting with people from other communities whom they perceive as judgemental. More research using an interactional approach [39–41], is required to understand the mechanisms at play.

Against the background of being uprooted from connections to place, the social-cultural rituals associated with weddings, funerals or making traditional beer serve to create a predictable social world, thus confirming group membership, and contributing to cohesion and solidarity [46]. Spiritual rituals have been identified as a significant strategy to deal with risks and adversities associated with loneliness [47,48]. Coping with loneliness included taking solitary actions in attempting to self-regulate, joining a group or keeping busy in everyday activities. Yet, activities in which older adults can engage often require financial resources, which are limited among older black people due to previous discriminatory policies and practices, thereby contributing to exclusionary impacts [22].

We have much to learn about loneliness of those who are among the most marginalized of older adults. Is their loneliness more powerfully negative than the ‘perceived discrepancy between the desired and achieved level of social’ relations’ [49, p. 31] which is the basis of widely used contemporary European definitions? How do the three components of loneliness found in this study: loss of intimate relationships; not being able to interact with people; impact of painful interactions relate to the social and emotional components of loneliness found in research from the global north?

Importantly, we have been unable to address possible gender differences in loneliness. Findings from the large European body of loneliness research indicate that higher proportions of women than men are lonely and that while much of women’s emotional loneliness comes from loss of their intimate partner, men are more likely to experience lack of satisfying social relations (e.g. [17, 49–49]). Our exploration of loneliness among older black South African women and knowledge of men’s relationship histories suggests that we have much to learn about how loneliness might emerge from their patterns of developing, maintaining and losing social relationships.

## Conclusion

Loneliness is fundamentally important though differently experienced across settings. In the predominantly Western context, loneliness is conceptualised as an unpleasant experience arising from a lack of sufficient quality personal relationships. Among older indigenous black South African women left vulnerable in terms of their group history of oppression, loneliness is regarded as an intensely unpleasant experience that manifests when there is no one to engage with. The findings of this study point to a previously unexplored understanding of loneliness resulting from disrupted community embeddedness.

## Paper context

Loneliness, associated with poor health outcomes among older individuals, prompted mostly Western regions to act. Older black South African women left vulnerable by lifelong exclusionary practices, experienced loneliness as intensely unpleasant and manifesting when there is no one to engage with, or when a community’s holding space is raptured. Acting on loneliness should include a community or social group perspective in re-connecting people to groups in a particular context they want to be connected.
